# Protective antibodies elicited by SARS-CoV-2 spike protein vaccination are boosted in the lung after challenge in nonhuman primates

**DOI:** 10.1126/scitranslmed.abi4547

**Published:** 2021-08-18

**Authors:** Joseph R. Francica, Barbara J. Flynn, Kathryn E. Foulds, Amy T. Noe, Anne P. Werner, Ian N. Moore, Matthew Gagne, Timothy S. Johnston, Courtney Tucker, Rachel L. Davis, Britta Flach, Sarah O’Connell, Shayne F. Andrew, Evan Lamb, Dillon R. Flebbe, Saule T. Nurmukhambetova, Mitzi M. Donaldson, John-Paul M. Todd, Alex Lee Zhu, Caroline Atyeo, Stephanie Fischinger, Matthew J Gorman, Sally Shin, Venkata Viswanadh Edara, Katharine Floyd, Lilin Lai, Seyhan Boyoglu-Barnum, Renee Van De Wetering, Alida Tylor, Elizabeth McCarthy, Valerie Lecouturier, Sophie Ruiz, Catherine Berry, Timothy Tibbitts, Hanne Andersen, Anthony Cook, Alan Dodson, Laurent Pessaint, Alex Van Ry, Marguerite Koutsoukos, Cindy Gutzeit, I.-Ting Teng, Tongqing Zhou, Dapeng Li, Barton F. Haynes, Peter D. Kwong, Adrian McDermott, Mark G. Lewis, Tong Ming Fu, Roman Chicz, Robbert van der Most, Kizzmekia S. Corbett, Mehul S. Suthar, Galit Alter, Mario Roederer, Nancy J. Sullivan, Daniel C. Douek, Barney S. Graham, Danilo Casimiro, Robert A. Seder

**Affiliations:** 1 Vaccine Research Center, National Institute of Allergy and Infectious Diseases, National Institutes of Health, Bethesda, MD 20814, USA.; 2 Ragon Institute of MGH, MIT, and Harvard, Cambridge, MA 02139, USA.; 3 Ph.D. program in Immunology and Virology, University of Duisburg-Essen, Essen, Germany.; 4 Ph.D. program in Virology, Division of Medical Sciences, Harvard University, Boston, MA 02138, USA.; 5 Centers for Childhood Infections and Vaccines, Children’s Healthcare of Atlanta and Emory University, Department of Pediatrics, Atlanta, GA, 30329, USA.; 6 Emory Vaccine Center, Emory University School of Medicine, Atlanta, GA 30329, USA.; 7 Yerkes National Primate Research Center, Atlanta, GA 30329, USA.; 8 Sanofi Pasteur, Marcy l'Etoile, France.; 9 Sanofi Pasteur, 38 Sidney Street, Cambridge, MA 02139, USA.; 10 Bioqual Inc., Rockville, MD 20850, USA.; 11 GSK, Wavre, Belgium.; 12 GSK, Rixensart, Belgium.; 13 Duke Human Vaccine Institute, Duke University, Durham, NC 27708, USA.

## Abstract

Adjuvanted soluble protein vaccines have been used extensively in humans for protection against various viral infections based on their robust induction of antibody responses. Here, soluble prefusion-stabilized spike protein trimers (preS dTM) from severe acute respiratory syndrome coronavirus 2 (SARS-CoV-2) were formulated with the adjuvant AS03 and administered twice to nonhuman primates (NHPs). Binding and functional neutralization assays and systems serology revealed that the vaccinated NHP developed AS03-dependent multifunctional humoral responses that targeted distinct domains of the spike protein and bound to a variety of Fc receptors mediating immune cell effector functions in vitro. The neutralizing 50% inhibitory concentration titers for pseudovirus and live SARS-CoV-2 were higher than titers for a panel of human convalescent serum samples. NHPs were challenged intranasally and intratracheally with a high dose (3 × 10^6^ plaque forming units) of SARS-CoV-2 (USA-WA1/2020 isolate). Two days after challenge, vaccinated NHPs showed rapid control of viral replication in both the upper and lower airways. Vaccinated NHPs also had increased spike protein–specific immunoglobulin G (IgG) antibody responses in the lung as early as 2 days after challenge. Moreover, passive transfer of vaccine-induced IgG to hamsters mediated protection from subsequent SARS-CoV-2 challenge. These data show that antibodies induced by the AS03-adjuvanted preS dTM vaccine were sufficient to mediate protection against SARS-CoV-2 in NHPs and that rapid anamnestic antibody responses in the lung may be a key mechanism for protection.

## Rapid response

Protein subunit–based vaccines have been used extensively for protection against viral infections. Here, Francica *et al*. tested a protein subunit vaccine for severe acute respiratory syndrome coronavirus 2 (SARS-CoV-2). The authors vaccinated nonhuman primates with soluble prefusion-stabilized spike trimers (preS dTM) plus the adjuvant AS03, an oil-in-water emulsion. The authors found that preS dTM plus AS03 induced robust antibody and cellular immune responses that protected nonhuman primates from disease when challenged with SARS-CoV-2. This rapid protection, with increases in antibodies specific to spike protein observable as soon as 2 days after infection, provides evidence of a critical anamnestic antibody response. Antibodies elicited by preS dTM vaccination are protective against SARS-CoV-2 in nonhuman primates.

## Teaser

Vaccination of nonhuman primates with SARS-CoV-2 spike trimers and AS03 adjuvant induces neutralizing antibodies sufficient to protect from disease.

## INTRODUCTION

The 2019 outbreak of coronavirus disease (COVID-19) caused by severe acute respiratory syndrome coronavirus 2 (SARS-CoV-2) has become a global pandemic with 181,734,810 infections and 3,936,298 deaths across 192 countries, as of 29 June 2021 (*1*). An effective prophylactic vaccine remains the most effective public health measure for controlling disease spread (*2*). To that end, two mRNA vaccines (*3*, *4*) have received emergency use authorization from the U.S. Food and Drug Administration (FDA) based on clinical efficacy of more than 90% in the United States. In addition, adenovirus-based vaccines have been approved for use in the European Union, United Kingdom (*5*), and Russia (*6*), and an inactivated virus vaccine is approved in China (*7*). Other protein-based vaccine candidates are currently in clinical testing (*8*). With the exception of the inactivated virus vaccines (*9*, *10*), these approved and clinical-phase candidate vaccines use only the coronavirus spike (S) protein as their immunogen.

Spike is a surface membrane–bound trimer that, by electron microscopy, gives viral particles a characteristic halo from which its family name, corona, is derived (*11*). It is a class I viral membrane fusion protein that exists in a metastable prefusion conformation and undergoes a marked structural rearrangement upon engagement of the receptor binding domain (RBD) with its receptor, angiotensin-converting enzyme 2 (ACE2) (*12*–*14*), ultimately leading to membrane fusion. It has been shown that antibodies directed against the RBD can neutralize incoming virus by preventing receptor recognition and, thus, entry (*15*–*19*). Because the RBD, as well as other regions such as the N-terminal domain (NTD), may contain neutralizing epitopes (*20*, *21*), the full-length spike is a preferred target antigen for vaccine development. On the basis of successful structure-based immunogen designs for SARS-CoV and Middle East respiratory syndrome virus vaccines (*22*, *23*), mutations have been introduced to block cleavage of S into S1 and S2 subunits and stabilize a region between the central helix and heptad repeat 1, giving rise to homogeneous S protein trimers in the prefusion conformation (*24*). This construct, referred to as proline-stabilized spike protein (S-2P), is the basis for several SARS-CoV-2 vaccine candidates being delivered by adenoviral vectors (*25*), displayed on nanoparticles (*26*), or encoded by mRNA (*3*, *27*, *28*).

In contrast to vectored gene delivery vaccine platforms, adjuvanted soluble protein vaccine formulations have been approved for clinical use against several viral infections such as hepatitis B and varicella zoster viruses (*29*–*31*) and have a long history of being used across all age groups. Soluble protein subunit vaccine candidates will likely require a potent adjuvant to elicit strong T and B cell responses (*32*). To date, several advanced vaccine candidates have been characterized for the magnitude, quality, and efficacy of the immune responses they elicit (*25*, *27*, *33*, *34*). Here, we have formulated a soluble S-2P–derived protein with the well-characterized adjuvant, AS03, an oil-in-water emulsion composed of squalene, polysorbate 80, and α-tocopherol. AS03 potently induces antibodies and has been shown to increase vaccine durability, promote heterologous strain cross-reactivity (*35*), and have dose-sparing effects (*36*–*40*). It was licensed for use in vaccines against pandemic influenza in Europe, with about 90 million doses administered (*36*, *40*–*42*). Therefore, in this study, AS03-adjuvanted soluble S-2P trimers were evaluated for nonhuman primate (NHP) immunogenicity and protection after SARS-CoV-2 challenge in advance of clinical trials. We performed a thorough characterization of humoral and cellular responses in the upper and lower respiratory tracts after vaccination and challenge. These studies establish that vaccine-induced antibody is sufficient for protection and highlight that rapid anamnestic antibody responses after challenge may be critical for control of lower airway viral replication.

## RESULTS

### Soluble spike trimers are immunogenic when adjuvanted with AS03

**Fig. 1.**
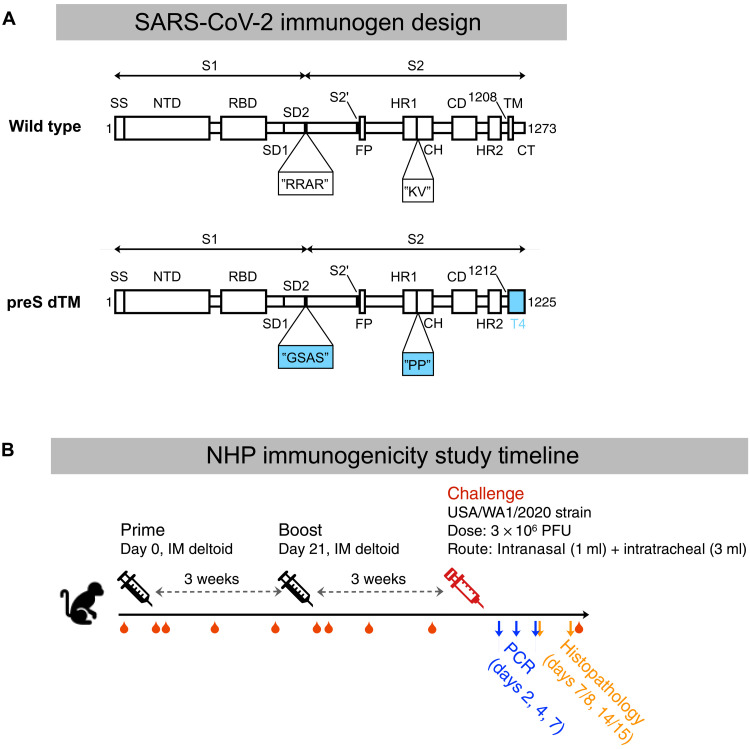
Vaccine design and study outline. (**A**) Schematic of the SARS-CoV-2 spike protein [adapted from (*24*)], preS dTM, with stabilizing mutations at the S1/S2 furin cleavage site and the heptad repeat region (amino acid sequences indicated by quotation marks); the transmembrane domain was replaced with a T4 trimerization domain. SS, signal sequence. SD, subdomain. FP, fusion peptide. HR, heptad repeat. CH, central helix. CD, connector domain. HR2, heptad repeat 2. TM, transmembrane domain. CT, cytoplasmic tail. (**B**) Schematic of NHP immunogenicity and challenge study. Immunizations were given at study weeks 0 and 3, intranasal and intratracheal SARS-CoV-2 challenge was performed at study week 6, blood draws are approximated by red droplets, and PCR and necropsy for histopathology approximated by arrows. IM, intramuscular.

To create a SARS-CoV-2 protein vaccine, the S-2P–stabilizing mutations were used as previously described (*24*); the trimer was then expressed as a soluble protein by replacing the transmembrane (TM) domain with a T4 foldon domain, which has been shown to assist in trimerization of type-1 membrane fusion proteins (Fig. 1A) (*43*, *44*). The resulting soluble trimeric protein immunogen is thus referred to as prefusion TM-deleted spike, or preS dTM, which was produced using a baculovirus expression system (*45*). PreS dTM trimers were then formulated by admixing with the oil-in-water emulsion, AS03. Rhesus macaques were immunized intramuscularly 3 weeks apart with or without AS03 to confirm its role for improving antibody responses. AS03 was critical for the induction of high-magnitude S-2P immunoglobulin G (IgG) binding and neutralization titers (fig. S1, A and B) and S-2P–specific IgA and IgG B cell responses (fig. S1, C to F). Systems serology was also performed to assess the quantitative and qualitative effector functions of vaccine responses induced by AS03. Antibodies were bound to a broad array of human antibody Fc receptors and enabled Fc-mediated effector functions such as phagocytosis and complement activation (fig. S1, G and H). AS03 strongly enhanced all Fc functions equally with no skewing to a particular receptor or function. Collectively, these data establish the critical role of the AS03 adjuvant for improving the magnitude and quality of antibody responses.

**Fig. 2.**
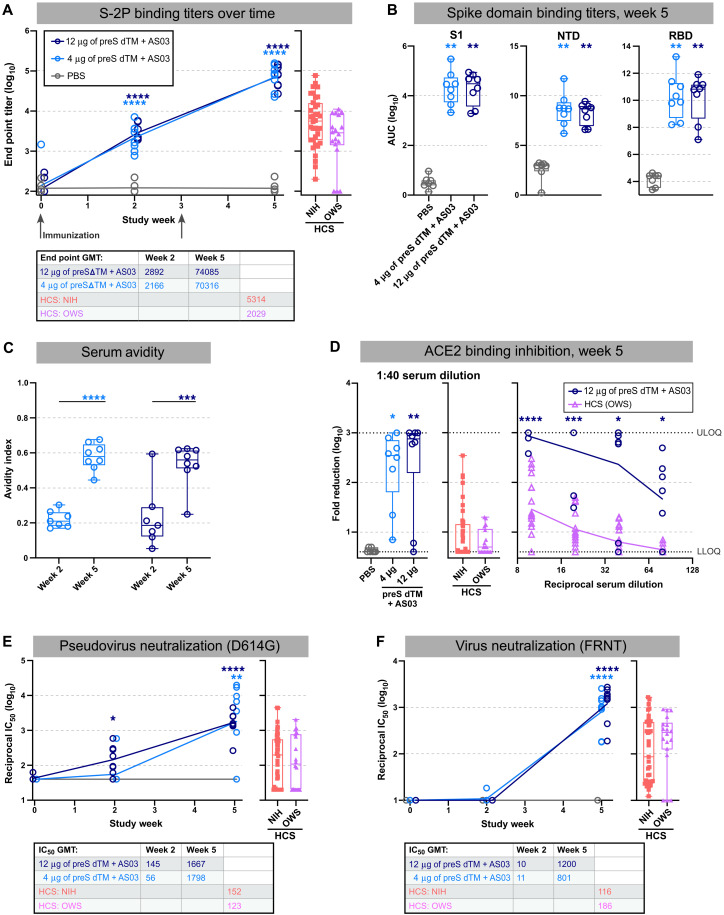
Vaccination with AS03-adjuvanted preS dTM induces spike protein–specific antibodies. Rhesus macaques were immunized with 4 or 12 μg of preS dTM adjuvanted with AS03 adjuvant at weeks 0 and 3. (**A**) End point binding titers after prevaccination (week 0) or after prime (week 2) and boost (week 5) were measured by ELISA (left). Immunization time points are indicated with gray arrows. End point titers from human convalescent sera (HCS) panels were measured as a comparison (*n* = 42, NIH; *n* = 18, OWS) (right). (**B**) Binding titers to the S1 domain, NTD, or RBD at week 5 were measured by Meso Scale Discovery (MSD) ELISA. AUC, area under the curve. (**C**) Avidity index at weeks 2 and 5. (**D**) Plasma inhibition of ACE2 binding to spike; week 5 vaccine dose response at 1:40 dilution (left graph) or over multiple dilutions (right graph) are shown. Dotted lines indicate upper limits of quantitation (ULOQ) and lower limits of quantitation (LLOQ). (**E** and **F**) Pseudovirus (E) or live virus (F) neutralization was measured over time after vaccination in NHPs or using HCS; 50% inhibitory concentration (IC_50_) values are plotted. Symbols represent individual animals, box plots indicate the median and interquartile range, and whiskers indicate minimum and maximum data points. Geometric mean values for binding (end point) and neutralization (ID_50_) titers are indicated in tables below each graph (GMT, geometric mean titer ). Asterisks indicate significance compared to the PBS control group as follows: *P < 0.05, **P < 0.01, ***P < 0.001, and ****P < 0.0001.

To study protective efficacy and perform a wider assessment of immunogenicity, rhesus macaques were immunized with 4 or 12 μg of AS03-adjuvanted preS dTM; phosphate-buffered saline (PBS) was administered as a negative control (Fig. 1B). Animals did not experience any abnormal body weight or temperature changes in response to vaccination (data file S1) nor were any other adverse events observed. Serum binding titers were detectible 2 weeks after the first immunization at concentrations that approximated those found in human convalescent donor sera (HCS) from two different benchmark cohorts; end point binding titers were increased from 2.9 × 10^3^ to 7.4 × 10^4^ after the second immunization in the high-dose group (Fig. 2A). There was no difference in dose response between the 4- and 12-μg dose groups. In terms of the breadth of binding, antibody responses were observed to the S1 region and, more specifically, to the RBD and NTD (Fig. 2B).

### Soluble spike trimers adjuvanted with AS03 induce neutralizing antibody responses

The next series of studies focused on functional antibody responses after AS03-adjuvanted preS dTM vaccination. We observed that the second immunization significantly improved serum avidity to S-2P in both dose groups (*P* = 0.0001; Fig. 2C). Sera from both vaccine dose groups also showed about a 100-fold higher competition with ACE2 for binding to the RBD compared to HCS (Fig. 2D). Inhibition of viral entry was next assessed using a pseudotyped reporter virus. Whereas neutralization was low or undetectable in most animals after the first immunization, reciprocal titers more than 10^3^ were achieved in nearly all animals after the boost (Fig. 2E). Similar results were seen with neutralization of live virus in a focus reduction neutralization titer (FRNT) assay, and these responses were generally 10-fold higher than those of HCS (Fig. 2F). On the basis of recent outbreaks of variant strains, we assessed neutralization against the B.1.1.7 “α” and B.1.351 “β” variants. There was a twofold decrease against the B.1.1.7 variant, and a 5- to 10-fold reduction against the B.1.351 variant (fig. S2).

### Soluble spike trimers adjuvanted with AS03 induce a mixed CD4 T cell response

**Fig. 3.**
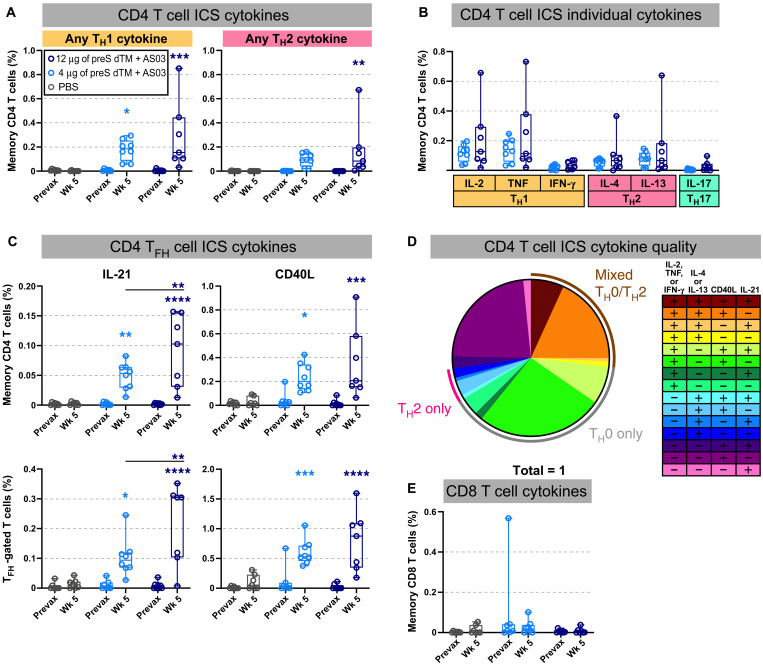
T cell responses are mixed after vaccination with AS03-adjuvanted preS dTM. T cell responses in rhesus macaques immunized with 4 or 12 μg of preS dTM adjuvanted with AS03 adjuvant at weeks 0 and 3. Cells taken before immunization (prevax) or at week (Wk) 5 were stimulated with an S1 peptide pool covering the spike protein and then assessed by intracellular cytokine staining. (**A**) Percent of memory CD4 T cells expressing any T_H_1 cytokine (IL-2, TNF, or IFN-γ; left) or any T_H_2 cytokine (IL-4 or IL-13; right). (**B**) Percent of memory CD4 T cells expressing the indicated cytokine. (**C**) Percent of CD4 T cells expressing the T_FH_ markers IL-21 (left) or CD40L (right) in all memory CD4 T cells (top) or the T_FH_ subset (bottom). (**D**) Proportion of memory CD4 T cells expressing any T_H_1 (IL-2, TNF, or IFN-γ), T_H_2 (IL-4 or IL-13), or T_FH_ (IL-21 or CD40L) markers by Boolean gating; week 5 responses from both vaccine dose groups are averaged. Pie arcs indicate the proportion of cells expressing any T_H_1 and T_H_2 cytokines in the same cell (brown arc); T_H_1 cytokines only (gray arc), or T_H_2 cytokines only (pink arc). (**E**) Percent of memory CD8 T cells expressing any T_H_1 cytokine (IL-2, TNF, or IFN-γ). Symbols represent individual animals, box plots indicate the median and interquartile range, and whiskers indicate minimum and maximum data points. Asterisks indicate significance compared to the PBS control group unless otherwise indicated as follows: *P < 0.05, **P < 0.01, ***P < 0.001, and ****P < 0.0001.

Because adjuvants also have an important effect on the magnitude and quality of CD4 T cells, we measured the frequency of spike-specific memory T helper (T_H_) cell subsets producing cytokines: T_H_1 [interleukin-2 (IL-2), tumor necrosis factor (TNF), and interferon-γ (IFN-γ)], T_H_2 (IL-4 and IL-13), T_H_17 (IL-17), and T follicular helper (T_FH_) cells [IL-21 and CD40 ligand (CD40L)] from peripheral blood mononuclear cells (PBMCs) by multiparameter flow cytometry. Two weeks after the boost (week 5), both T_H_1 and T_H_2 cytokines were detected (Fig. 3A). In assessing individual cytokines, the T_H_1 response was composed mostly of IL-2 and TNF with minimal IFN-γ production, indicative of a “T_H_0” phenotype (Fig. 3B) (*46*, *47*). Antigen-specific IL-21 production and CD40L expression were detected in both CD4 memory and T_FH_-gated PBMC subsets, supporting their role in the robust antibody responses induced after vaccination (Fig. 3C). To further analyze cytokine production on a single-cell basis, Boolean gating was used to show the various combinations of cytokines (Fig. 3D). More than ~89% were CD40L^+^, a sensitive marker for antigen-specific cells. Only 6.5% of cells produced only T_H_2 cytokines, whereas about 27% produced combinations of IL-2 or TNF, and IL-4 or IL-13, characterized as a mixed or “T_H_0” phenotype. CD8 T cell responses were largely undetectable (Fig. 3E).

### Soluble spike trimers adjuvanted with AS03 protect NHPs from high-dose SARS-CoV-2 challenge

**Fig. 4.**
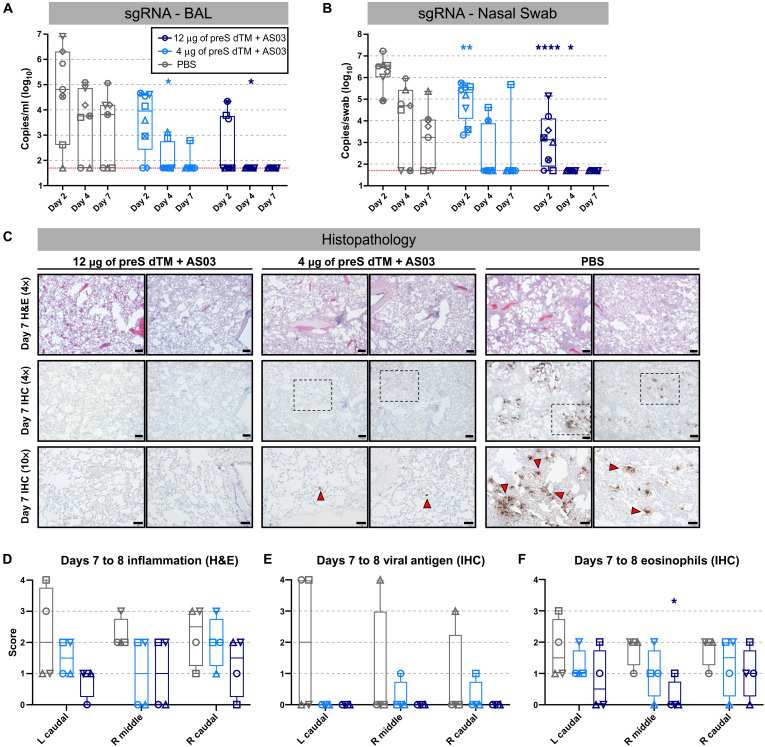
Vaccination with AS03-adjuvanted preS dTM protected NHP from SARS-CoV-2 challenge. Rhesus macaques immunized with 4 or 12 μg of AS03-adjuvanted preS dTM were challenged with 3 × 10^6^ PFU of SARS-CoV-2 by the intranasal and intratracheal routes. SARS-CoV-2 subgenomic RNA (sgRNA) in BAL (**A**) and nasal swabs (**B**) was measured at 2, 4, and 7 days after challenge. Symbols represent individual animals; bars indicate group geometric means. Red dotted lines indicate lower limit of quantitation. (**C**) Histopathological analysis at 7 days after challenge. Representative images from lung sections from two animals per group analyzed by hematoxylin and eosin (H&E) staining for inflammation (top; ×4 magnification) or IHC staining for viral antigen (middle; ×4 magnification; bottom; ×10 magnification). Scale bars, 200 and 100 μm for ×4 and 10× magnifications, respectively. Red arrowheads indicate foci of viral antigen. (**D** to **F**) Quantification of histopathology for four animals at days 7 to 8 after challenge. Scores indicating inflammation from H&E staining (D), viral antigen (E), or eosinophil infiltration (F). Symbols represent individual animals, box plots indicate the median and interquartile range, and whiskers indicate minimum and maximum data points. Asterisks indicate significance compared to the PBS control group as follows: *P < 0.05, **P < 0.01, and ****P < 0.0001.

Prior NHP vaccine studies (*25*, *48*) have used varying doses of the USA-WA1/2020 isolate ranging from 10^4^ to 10^6^ plaque forming units (PFU) for nasal and intratracheal challenge. In addition, passaging of the USA-WA1/2020 isolate has led to mutations in the furin cleavage site that can limit pathogenicity and results in variation of the amount and duration of infection in NHPs. Thus, in this study, NHPs were challenged 3 weeks after the boost with a new sequence-validated stock of the USA-WA1/2020 isolate that was administered at a high dose of 3 × 10^6^ PFU of SARS-CoV-2 given intranasally and intratracheally. Lower airway protection was assessed using subgenomic RNA (sgRNA), as a quantitative metric of replicating virus (*49*) in bronchoalveolar lavage (BAL) (Fig. 4A). At day 2, six of seven (86%) PBS control animals had detectable sgRNA compared to six of eight (75%) and three of eight (38%) in the 4- and 12-μg dose groups, respectively. By day 4, five of seven (71%) of PBS control animals were positive, but sgRNA was significantly reduced to two of eight (25%; *P* = 0.0442) or zero of eight (0%; *P* = 0.0201) in the 4- and 12-μg vaccine dose groups, respectively. By day 7, sgRNA was detectable in four or seven (57%) PBS controls but only 1 of 16 (6%) vaccinated animals. To assess the upper airway protection, sgRNA was quantified in nasal swab extracts (Fig. 4B). Both vaccine groups showed significant sgRNA reduction on day 2 (4-μg dose: *P* = 0.0098; 12-μg: *P* < 0.0001). By day 4, five of seven (71%) PBS controls had detectible sgRNA (10^4^), whereas only two of eight (25%) and zero of eight (0%) vaccinated NHPs had detectable sgRNA in the 4- and 12-μg dose groups, respectively. Thus, AS03-adjuvanted preS dTM provided substantial protection in the upper (Fig. 4A) and lower (Fig. 4B) airways from SARS-CoV-2 challenge.

To further substantiate vaccine protection, lung tissue was analyzed for viral antigen, inflammation, and eosinophil infiltration in half of the animals in each group 7 days after challenge (Fig. 4, C to E). Viral antigen was detected in at least one lobe of three of four PBS control animals. In contrast, antigen was undetectable in the high-dose vaccinated animals and had only limited detection in two of the low-dose vaccinated animals. Vaccination at either dose did not result in increased tissue inflammation (Fig. 4D) nor an increase in eosinophils after challenge (Fig. 4F).

### SARS-CoV-2 challenge boosted antibody titers in the lungs of vaccinated NHPs

To further investigate how T cells or antibodies may have influenced protection in the respiratory tissues, we assessed T cell responses in the BAL and PBMC and antibody responses in the serum, BAL, and nasal washes at various time points after challenge. Compared to the peak T cell responses after the second immunization at week 5 when restimulated with S peptides (Fig. 3, A and B), memory PBMC CD4 and CD8 T cell responses were largely unchanged 7 to 14 days after challenge (fig. S3, A and B). However, in BAL samples, spike-specific IL-2, IFN-γ, and IL-13 recall responses were increased in the vaccinated groups compared to week 5, but not in the PBS controls (fig. S3, C and D). To assess the primary T cell response to infection, cells were restimulated with peptides to nucleoprotein, which is not present in the vaccine. Here, we noted that the SARS-CoV-2 challenge induced a strong T_H_1 response in the BAL but not in PBMCs by day 14 that was specific to the PBS control animals (fig. S3, E and F). These data suggest that vaccine-elicited immune responses controlled the infection before a detectable primary T cell response could be generated in BAL or PBMCs.

**Fig. 5.**
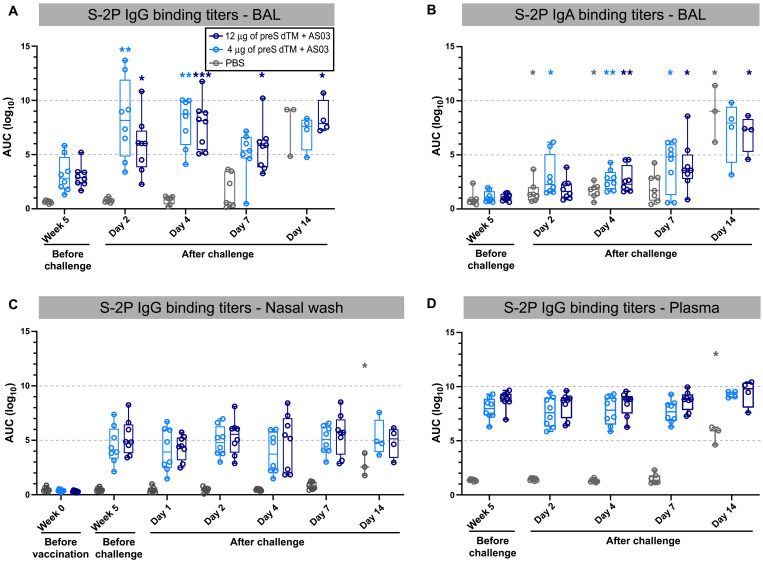
Anamnestic antibody responses are initiated in the lung after SARS-CoV-2 challenge. BAL supernatant was collected before challenge (week 5) and on days 2, 4, 7, and 14 after SARS-CoV-2 challenge. (**A** and **B**) S-2P IgG (A) and IgA (B) binding titers in BAL samples were calculated. (**C** and **D**) S-2P IgG binding titers in nasal washes (C) and plasma (D) taken before and after challenge were measured. Symbols represent individual animals, box plots indicate the median and interquartile range, and whiskers indicate minimum and maximum data points. Asterisks indicate significance compared to the PBS control group as follows: *P < 0.05, **P < 0.01, and ***P < 0.001.

Because vaccinated animals showed no detectable primary N-specific T cell response to the challenge in BAL or PBMCs, it suggested rapid control of infection by the vaccine in the airways, which we hypothesized might be mediated by antibodies. To assess this, we performed a kinetic analysis of antibody responses in BAL and nasal washes up to 2 weeks after challenge. S-2P IgG binding titers were significantly increased in the BAL from vaccinated animals just 2 days after challenge and remained higher than the control animals through day 7 (4-μg dose: *P* = 0.0057; 12-μg dose: *P* = 0.0126; Fig. 5A). In contrast, IgA and IgG responses to the challenge developed only by day 14 in the PBS control animals, consistent with the kinetics of a primary response (Fig. 5, A and B). This anamnestic response in the vaccinated animals was specific to the BAL, as there was no increase in S-2P IgG titers in nasal washes (Fig. 5C) or sera (Fig. 5D) after challenge. The primary antibody response was evident in blood and upper and lower airways by day 14 in the PBS control animals. On the basis of the rapid anamnestic response in BAL in the vaccine groups, we next determined whether this was specific to S-2P antibodies or whether the challenge was causing a general increase in IgG. There was an increase in total IgG in BAL of vaccinated animals at 2 days after challenge, continuing through day 4 and decreasing by day 7, whereas PBS control animals had a smaller increase on day 4 only (fig. S4A). We next assessed whether other antibody specificities were increased in BAL after challenge. Because all NHP used in our studies have had earlier vaccination against measles, we assessed measles antibody titers in BAL. Consistent with increases in spike and total IgG titers in BAL, vaccinated animals similarly showed a significant increase in measles antibodies on days 2 and 4 compared to prechallenge, and several PBS control animals also showed increasing measles antibodies on day 4 (day 2, 4-μg dose: *P* = 0.0122; 12-μg dose: *P* = 0.0245; day 4, 4-μg dose: *P* = 0.0475; 12-μg dose: *P* = 0.0262; fig. S4B). Last, to assess whether this increase in total IgG could be due to increased general transudation into the lung from the serum, we assessed the serum protein albumin concentrations in BAL. Albumin concentrations in BAL were not increased in the vaccinated or PBS control animals 2 or 4 days after SARS-CoV-2 challenge (*P* > 0.05; fig. S4C). These data show that SARS-CoV-2 challenge leads to a rapid and transient local increase in IgG that occurs earlier in vaccinated animals.

### Vaccine-induced IgG is sufficient to confer protection from SARS-CoV-2 challenge in hamsters

**Fig. 6.**
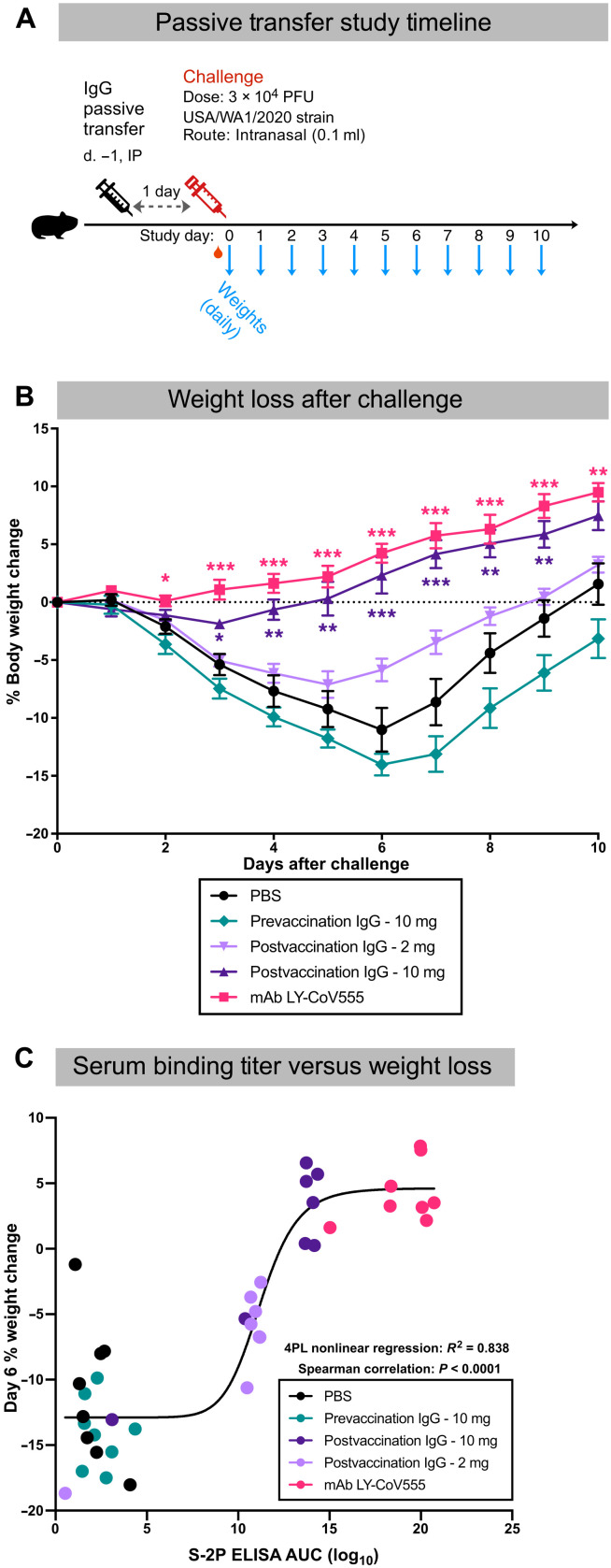
Passively transferred IgG from vaccinated NHP protected hamsters from SARS-CoV-2 challenge. Total IgG was isolated from pooled week 6 sera from rhesus macaques immunized with 3 μg of AS03-adjuvanted preS dTM. Ten or 2 mg of total IgG was transferred to hamsters; 10 mg of IgG from before (pre-) vaccination or PBS was transferred as a negative control for protection; mAb Ly-CoV555 (10 mg/kg) was transferred as a positive control. Animals were then challenged 1 day later with SARS-CoV-2; body weight was recorded daily, and oral swabs were taken for PCR on days 2, 4, and 7. (**A**) Passive transfer study timeline. (**B**) Daily change in body weight after challenge. Lines depict group mean body weight change from day 0; error bars represent SEM. (**C**) The correlation between serum S-2P binding titers and percent weight loss on day 6 after SARS-CoV-2 challenge is shown. Curve depicts a four-parameter logistic fit of the data. Symbols represent individual animals; whiskers indicate SEM. Asterisks indicate significance compared to the PBS control group at each time point: *P < 0.05, **P < 0.01, and ***P < 0.001.

On the basis of the high antibody and neutralizing titers in the blood and rapid anamnestic antibody responses in the BAL after challenge, we hypothesized that IgG was mediating protection. To directly assess whether vaccine-induced antibodies were sufficient to mediate protection, NHP IgG was purified from pooled plasma 3 weeks after the second vaccination just before the challenge and passively transferred to hamsters (Fig. 6A). A total of 10 or 2 mg of total IgG per animal from AS03-adjvuanted preS dTM–vaccinated NHPs or from animals before vaccination as a negative control was administered to eight individual hamsters per group. This resulted in about 125 and 25 mg of IgG/kg body weight for the 10- and 2-mg dose groups, respectively (fig. S5A). PBS was administered to an additional group as a negative control, and the highly potent and clinically approved SARS-CoV-2 monoclonal antibody (mAb), LY-CoV555 (*50*), was administered to another group at 10 mg/kg as a positive control. Just before challenge, serum titers were confirmed by S-2P binding enzyme-linked immunosorbent assay (ELISA) (fig. S5, B and C) and pseudovirus neutralization (data file S2) in all but two animals, which were subsequently excluded. The LY-CoV555 mAb recipient animals had higher binding and pseudovirus neutralization titers than those that received polyclonal postvaccination IgG. Hamsters that received only PBS before SARS-CoV-2 challenge lost an average of 10 to 15% of their body weight at day 6, a primary outcome measure of disease progression (Fig. 6B and data file S3). Hamsters that received 10 mg of postvaccination IgG had little weight loss and gained weight at a rate almost equivalent to that of the LY-CoV555 recipient hamsters. This protection was dose dependent, because the animals that received 2 mg of postvaccination IgG showed weight loss of about 7% by day 6. Last, individual animal serum S-2P binding titers were strongly correlated with body weight change, confirming the effect of IgG in protection from challenge (*P* < 0.0001; Fig. 6C). Together, these data show that the AS03-adjuvanted preS dTM vaccine elicited IgG sufficient to mediate protection in vivo against SARS-CoV-2 infection.

## DISCUSSION

Although SARS-CoV-2 mRNA- and adenovirus-based vaccine candidates have been authorized for emergency use in various countries, adjuvanted protein vaccines provide an additional vaccine platform to prevent disease that could be broadly useful in all age groups based on their long history and safety record with other viral infections. In this study, an AS03-adjuvanted soluble prefusion S protein vaccine formulation produced by Sanofi Pasteur and GlaxoSmithKline was evaluated in NHPs in advance of clinical trials. The primary aims of this study were to evaluate immunogenicity after vaccination and to assess protection after SARS-CoV-2 challenge. A secondary aim was to investigate potential mechanisms of protection. These studies provide new insights into protective antibody responses, most notably in the lung, which are critical for understanding how vaccines limit disease, a primary end point in all clinical trials.

By comparing the immune response to preS dTM formulations with and without AS03, it is clear that AS03 is critical for the induction of protective antibody responses, as has been previously observed with influenza (*36*, *37*, *51*, *52*) and respiratory syncytial virus (RSV) (*53*). The CD4 T cell responses to spike were primarily T_H_0, given the relative limited IFN-γ production (*46*), and T_H_2. In mouse studies, IL-4 and IL-13 production was also observed after vaccination with an inactivated influenza/AS03 formulation (*54*). Previous human studies of AS03 with hepatitis B surface antigen (*55*) and influenza hemagglutinin (*56*) have demonstrated strong IL-2 and TNF production with lower IFN-γ responses. However, IFN-γ responses were recently documented in humans with AS03 and a similar antigen (SCB-2019) (*57*), suggesting that the CD4 profile might differ depending on the species and the antigen. On the basis of mouse and other animal models, vaccine-induced T_H_2 responses have been proposed to contribute to enhanced respiratory disease (ERD) (*58*–*60*), as was observed in children given inactivated measles (*61*) and RSV (*62*) vaccines. Similarly, SARS vaccines formulated with the T_H_2-skewing adjuvant, alum, have been reported to induce immunopathology after challenge in mice, including eosinophilia (*63*). Other studies suggest ERD is driven by nonfunctional and poorly matured antibodies (*58*, *64*, *65*). Regardless of the mechanism, and in contrast to those findings, after SARS-CoV-2 challenge, there was limited evidence of viral infection, and there was no evidence of increased inflammation and eosinophil infiltration in lung tissue from vaccinated animals compared to PBS controls, indicating no enhanced disease.

A major focus of this study was to fully characterize the magnitude, quality, and location of antibody responses. NHP models have been used extensively for COVID-19 vaccine development and share characteristics of mild human disease. Thus, a major advantage of using NHPs is the ability to analyze immune responses in the mucosa of the upper and lower airways. Regarding the magnitude of antibody responses, pseudovirus and live virus neutralization titers were above 10^3^ in most animals 2 weeks after the second immunization of AS03-adjuvanted preS dTM. These responses are comparable to a prior study using the same assays after 100 μg of the mRNA nanoparticle vaccine, mRNA-1273 (*27*), and are superior to neutralization titers in sera from convalescent humans.

Regarding the quality of antibody responses, we observed strong Fc receptor–binding and Fc-mediated functional activity, which has been reported to correlate with protection in the NHP model (*66*). Other animal and human studies have also shown the importance and contribution of non-neutralizing antibody titers and effector functions to protection (*25*, *67*–*69*). However, we note that both neutralizing antibodies and Fc-functional responses themselves correlate, therefore making it difficult to interpret the role of either in providing protection.

In terms of the location of antibody responses, AS03-adjvuanted preS dTM was able to rapidly reduce viral replication in both the upper and lower airways by day 2, with no detectable virus in any of the animals in the high-dose group by day 4. Comparing these results to other vaccines is difficult based on differences in the challenge dose and virulence of the virus stock. Here, we have used a high-challenge dose of 3 × 10^6^ PFU, which is 5- to 200-fold higher than the doses used to evaluate mRNA-1273 (*27*), Ad26.COV2-S (*25*), ChAdOx1 nCoV-19 (*34*), and NVX-CoV2373 (*33*) but similar to that used for BNT162b2 (*48*). The relatively higher challenge dose used in this study may, in part, explain why there was no complete reduction in viral titers at day 2, as was observed with other vaccines tested in NHPs using lower challenge doses. Similarly, we observed nasal swab titers of ~2 × 10^6^ sgRNA copies/ml in the control animals at day 2, which were 10- to 50-fold higher than previously reported in other NHP studies by us and others (*27*, *34*), likely reflecting both the more virulent challenge and improved sgRNA extraction and quantitation methods.

Because substantial protection was conferred by vaccination with AS03-adjuvanted preS dTM, it was critical to investigate the potential mechanisms for this effect. Consistent with data from most protein subunit vaccine studies in humans with AS03 and other adjuvants, CD8 T cell responses were undetectable. Protein vaccines with clinically approved adjuvants have historically been limited for cross-presentation and inducing CD8 T cell responses. Furthermore, AS03 induces little type I IFN production, which is often required for cross-presentation to generate CD8 T cells. These data suggest that, with this vaccine and adjuvant, CD8 T cells would have a limited role in primary, rapid protection. In contrast, mRNA-based vaccines mRNA-1273 (*27*) and BNT162b2 (*47*) induce detectible CD8 T cell responses and antibody responses. Their role in mediating protection in NHP models or in humans remains unknown. CD8 T cell intracellular cytokine responses induced by AD26.COV2.S were reported not to correlate with protection in NHP studies (*66*). In regard to CD4 T cells, antigen-specific CD4 T cells were composed of T_H_0 and T_H_2 responses. T_FH_ cells that expressed CD40L were readily detected and are important for generating robust antibody responses.

On the basis of the early control of infection seen by day 2, it is likely that antibodies had an important role in neutralizing replicating virus. The demonstration that neutralizing antibodies could protect against viral challenge has been shown in correlative analyses with other SARS-CoV-2 vaccine candidates (*27*, *67*). Moreover, prior studies have shown a protective effect from passively transferred convalescent sera to hamsters (*70*) and NHPs (*66*). In this study, we showed that passive transfer of vaccine-elicited IgG could protect against SARS-CoV-2 challenge in hamsters, providing direct evidence that antibodies are sufficient to mediate protection. An additional and key finding of the study that substantiates a role for antibodies was the rapid increase in IgG responses in the lung as early as 2 days after challenge in NHPs. Mucosal antibody responses to vaccination have been well documented, including response to polio (*71*–*73*), influenza (*74*, *75*), and RSV (*76*, *77*) vaccines. Moreover, neutralizing BAL responses have been observed in humans infected with SARS-CoV-2 in the initial days after symptom onset (*78*). However, vaccine-specific anamnestic lung antibody responses after challenge are not well described in the literature. This rapid increase in spike IgG appears specific to the BAL compartment and was not observed in the upper airway or serum after challenge. A notable finding was that there was also a transient increase in total IgG titers and measles antibody titers from prior vaccination in the BAL at day 2. However, albumin concentrations were not increased in BAL. Together, these data suggest that the increase in antibodies may not be due to a general transudation of proteins from increased vascular leakage or bulk vesicular transport. We speculate that this increase in total and antigen-specific IgG could occur through a general activation of memory B cells in the lung, perhaps directly from Toll-like receptor sensing of viral RNA (*79*) or through bystander activation from activated S-specific CD4 T cells. Together, this could explain why IgG titers were higher in vaccinated animals on day 2, whereas PBS controls showed a smaller increase in total and measles IgG only on day 4. Although antibody secreting cell (ASC) enumeration was not possible in this study, future studies will examine changes in lung-resident ASCs and investigate whether an increase in BAL antibodies is specific to this vaccine or is generalizable to other vaccine platforms. Nevertheless, these data highlight the potential role of ASCs in contributing to control of viral infections in the lung. Last, it will be important to determine whether these findings of anamnestic responses in the lungs after challenge are observed with other vaccines or after a secondary infection.

There are several limitations to our study. This study provides strong evidence for the protective role of neutralizing antibody responses elicited by recombinant spike protein formulated in AS03 adjuvant. We also show data that anamnestic antibody responses in the vaccinated animals are rapidly induced in the lung after infectious challenge. Thus, a major outstanding question is the mechanism by which such responses are induced in the lung. Future studies will focus on whether innate immunity induced by the viral challenge is leading to activation of resident B cells or plasma cells in the lung leading to the rapid production of antibody. Another question not directly addressed in this study is whether T cells have a direct effector role in mediating protection. Because there was little induction of antigen-specific CD8 T cells, it was unlikely that they had a role in primary protection. Investigating a direct role for antigen-specific CD4 T cells in control of viral load independent of helping antibody responses would have required their depletion before challenge, which was not assessed in this study. Last, although this study benchmarked serum responses to two panels of HCS, our responses were not reported using World Health Organization standards for defining binding antibody responses to WA-1 spike protein in international units. These standards were not available when our analysis was performed. However, we would note that serum titers here were about 1 log higher than average HCS responses, indicating strong immunogenicity relative to convalescent responses.

In conclusion, this report highlights that potent serum antibody responses are induced by soluble S trimers formulated with the oil-in-water emulsion adjuvant AS03, which conferred protection in the upper and lower airways of NHPs after SARS-CoV-2 challenge. This vaccine induced IgG responses that were sufficient to protect from SARS-CoV-2 challenge in hamsters. The rapid anamnestic response observed in the lower airway of vaccinated NHPs likely contributed to protection at this site. These data support the clinical development of the AS03-adjuvanted preS dTM vaccine for limiting SARS-CoV-2 infection and protecting against COVID-19 disease.

## MATERIALS AND METHODS

### Study design

Animals were assigned to groups to achieve an even distribution of animal age, weight, and gender. Group size was calculated to give at least 90% power to detect a 1.95 log reduction in viral RNA compared to the control group, assuming up to four comparisons to control; these calculations were based on deviation estimated from (*67*), a study also performed at Bioqual Inc. Animal vaccine group assignments were not blinded to those collecting data. Raw data are presented in data file S4.

### Immunogen

The SARS-CoV-2 recombinant vaccine candidate consists of purified recombinant prefusion spike (S) protein [SARS-CoV-2 prefusion-stabilized S with the TM region deleted (preS dTM)] adjuvanted with AS03. The preS dTM was produced from a Sanofi Pasteur proprietary cell culture technology based on the insect cell–baculovirus system, referred to as the Baculovirus Expression Vector System. The preS dTM sequence was designed on the basis of the Wuhan YP_009724390.1 strain S sequence but modified to improve the conformation, stability, and trimerization and to facilitate the purification. The modifications comprise mutation of the S1/S2 furin cleavage site, introduction of two proline mutations in the C-terminal region of S2 domain, deletion of the TM and cytoplasmic region, and replacement by the T4 foldon trimerization domain (*24*). Briefly, the modified sequence was cloned into a baculovirus transfer plasmid, which was then used to generate a recombinant baculovirus containing the gene of interest. The recombinant baculovirus was first amplified in expresSF+ insect cells before infecting a large-scale expresSF+ insect cell culture in suspension. After incubation, the recombinant protein was purified from the supernatant using several affinity and chromatography columns, as previously described (*45*). On the basis of an ACE2 binding assay, the preS dTM used in the study were quantified at 4 and 12 μg for a total protein content of 5 and 15 μg for the low and high doses, respectively.

### Adjuvant and formulation

AS03 is an adjuvant system composed of α-tocopherol, squalene, and polysorbate 80 in an oil-in-water emulsion (*80*). Vaccine doses were formulated by diluting the appropriate dose of preS dTM with PBS to 250 μl, then mixing with 250 μl of AS03, followed by inversion five times for a final volume of 500 μl. Each dose of AS03 contains 11.86 mg of α-tocopherol, 10.69 mg of squalene, and 4.86 mg of polysorbate 80 (Tween 80) in PBS.

### Animals, immunizations, challenges, and sampling

Rhesus macaques were randomized into groups of eight based on age and body weight; each group had two females and six males, except for the PBS control group, which only had five males. All animals had a history of measles vaccination. SARS-CoV-2 vaccine formulations were administered by intramuscular injection into the right deltoid for both immunizations, 3 weeks apart. Whole blood was collected weekly into EDTA-containing tubes. PBMC and plasma were then collected from whole blood after Ficoll purification. For SARS-CoV-2 challenge, virus was obtained from Operation Warp Speed: strain 2019-nCoV/USA-WA1/2020, lot no. 70038893; Biodefense and Emerging Infections Research Resources Repository (BEI Resources), catalog no. NR-53780. Virus was inoculated intranasally (0.5 ml per nostril) and intratracheally (3 ml) for a total of 3 × 10^6^ PFU per animal. BAL sampling was performed 5 weeks after vaccination and 2, 4, and 7 days after challenge. Nasal swabs for sgRNA polymerase chain reaction (PCR) were taken 5 weeks after vaccination and 2, 4, and 7 days after challenge; nasal washes (5 ml of PBS) were collected at weeks 0 and 5 and 1, 2, 4, 7, and 14 days after challenge. Half the animals in each group were necropsied at days 7 and 14 after challenge, where the lung tissue was collected for histopathology.

In a separate study, four groups of six rhesus macaques were similarly vaccinated, but with lower doses of 1.3 and 3.9 μg of preS dTM + AS03, 3.9 μg of preS dTM without AS03, or PBS alone. Animals were then boosted at week 3 with 2 and 6.1 μg of preS dTM + AS03, 6.1 μg of preS dTM without AS03, or PBS alone. Immunogenicity data from this study are found in fig. S1. IgG was purified from animals given 3 μg of preS dTM + AS03 either before immunization or 3 weeks after the second immunization for use in passive transfer to hamsters as shown in Fig. 6.

For passive transfer studies, 6- to 8-week-old golden Syrian hamsters were randomized into groups of eight based on weight, each group having four males and four females. Total IgG was purified from pooled NHP plasma using the Protein G Sepharose 4 Fast Flow resin (Cytiva) according to the manufacturer’s instructions. The eluted protein was dialyzed against 1× PBS (pH 7.4) (Gibco) and concentrated to 10 mg/ml using the Amicon Ultra centrifugal filter (Millipore Sigma). Concentration was determined using the NanoDrop One Microvolume Ultraviolet-Visible Spectrophotometer (Thermo Fisher Scientific). IgG was passively transferred in a 1-ml volume by intraperitoneal injection 1 day before challenge. SARS-CoV-2 challenge virus (strain 2019-nCoV/USA-WA1/2020, lot no. 70038893; BEI resources, catalog no. NR-53780) was introduced intranasally at a dose of 3 × 10^4^ PFU administered in a final volume of 100 μl and split between each nostril. Body weight and clinical observations were made daily; serum was sampled just before challenge.

### Ethics statement

Macaques were housed at the National Institutes of Health (NIH) (for immunizations) and Bioqual Inc. (for challenge); hamsters were housed at Bioqual Inc. All animals were cared for in accordance with the American Association for Accreditation of Laboratory Animal Care standards in accredited facilities. All animal procedures were performed according to protocols approved by the Institutional Animal Care and Use Committees of the National Institute of Allergy and Infectious Diseases, NIH and Bioqual Inc. NHP studies were performed under NIH animal study protocol no. VRC-20-870; hamster studies were performed under animal study protocol #VRC-20-872.

### Human convalescent sera

Two panels of samples from human patients who had recovered from SARS-CoV-2 disease were used in parallel. The first panel referred to as “NIH” has been described previously (*27*). In addition, an 18-sample panel collected by Operation Warp Speed and distributed by Battelle and BEI Resources was also used, referred to here as “OWS”. Informed consent was obtained from all participants. Participants had a history of laboratory-confirmed SARS-CoV-2 infection before they provided serum.

### Enzyme-linked immunosorbent assay

Antibody titers to various SARS-CoV-2–derived antigens were assayed as previously described (*27*). Briefly, end point binding titers were measured by standard sandwich ELISA using S-2P. Binding to SARS-CoV-2 S1, RBD, and NTD spike subdomains was performed using Meso Scale Discovery (MSD) ELISA (*27*), using biotinylated subdomain proteins prepared as described previously (*81*). Similarly, the MSD ELISA was used to measure titers after challenge and to perform high-throughput batch analyses of multiple time points, where area under the curve (AUC) is reported. By performing an analysis of serially diluted sera, we have found that a threefold dilution of sera results in about 2 log reduction in AUC binding titer; similarly, a 10-fold dilution of sera results in a 3 log reduction in AUC.

### IgG quantification

Total IgG antibody titers were quantitated by using the Human IgG ELISA^BASIC^ Kit (ALP) (Mabtech) following the manufacturer’s instructions. Samples were read using an MSD plate reader (Sector Imager 600). Antibody titers to measles were quantitated by using the Monkey Anti-Measles IgG ELISA Kit (Alpha Diagnostics International) following the manufacturer’s instructions. The optical density (OD) of each well was read at 450 nm using the SpectraMax Paradigm Multi-Mode Microplate Reader (Molecular Devices). Albumin concentrations were measured by bead-based single-plex assay using Luminex. The albumin analyte was selected and measured using the MILLIPLEX MAP Human Kidney Injury Magnetic Bead Panel 2 (Millipore Sigma) following the manufacturer’s instructions. Fluorescence data were collected on MAGPIX with Bio-Plex ManagerTM MP software (Bio-Rad). ACE2 binding inhibition was also performed via MSD 384-well, 4-Spot Custom Serology SECTOR plates precoated with RBD; plasma was applied at a starting dilution of 1:10 followed by 10-fold serial dilutions. Binding was detected with SULFO-TAG–labeled ACE2 (Meso Scale Diagnostics).

### Avidity analysis

For avidity analyses, plasma samples were heated at 56°C for 45 min to complement-inactivate and reduce potential risk from any residual virus and immediately used or stored at −80°C for later use. Ninety-six–well plates (Nunc MaxiSorp, Thermo Fisher Scientific) were coated with 100 μl of SARS-CoV-2 S-2P (1 μg/ml) in 1× PBS for 16 hours at 4°C. Plates were washed three times in washing buffer [1× PBS and 0.2% Tween 20 (pH 7.4)] using a BioTek 405 microplate washer and blocked with 200 μl of blocking buffer [0.14 M NaCl, 0.0027 M KCl, 0.05% Tween 20, and 0.010 M PO_4_^3−^ (pH 7.4)] for 2 hours at room temperature and washed three times. Plasma samples were serially diluted (starting dilution of 1:100 and fourfold dilutions) in blocking buffer, and 100 μl was transferred to the plates. After 1 hour of incubation, plates were washed, and half of the samples were then incubated with 100 μl of 1× PBS. The other half of the paired samples were treated with 100 μl of 1.0 M sodium thiocyanate solution (NaSCN; Sigma-Aldrich) for 15 min at room temperature and washed six times. Plates were incubated for 1 hour with 100 μl of goat anti-human IgG (H+L; catalog no. PA1-8463) or goat anti-monkey IgG (H+L; catalog no. A18811) secondary antibody conjugated to horseradish peroxidase (Thermo Fisher Scientific) in a blocking buffer at 1:10,000 or 1:4000 dilution, respectively. Plates were washed three times and developed by addition of 100 μl of the SureBlue 3,3′,5,5′-tetramethylbenzidine (TMB) Microwell Peroxidase Substrate (1-Component; SeraCare, catalog no. 52-00-01) for 10 min. The reaction was quenched by addition of 100 μl of 1 N of H_2_SO_4_, and absorbance was measured at a test wavelength of 450 nm and reference wavelength of 650 nm using SoftMax Pro software version 6.5 on a SpectraMax Paradigm microplate reader (Molecular Devices). The avidity index (AI) was calculated using the ratio of the NaSCN-treated serum dilution giving an OD of 0.5 to the PBS-treated serum dilution giving an OD of 0.5 after five point logistic curve fitting in GraphPad Prism. Reported AI is the average of two independent experiments, each containing duplicate samples. Samples with an OD < 0.5 could not be interpolated and were excluded from analysis.

### Pseudoviral neutralization

To produce SARS-CoV-2 pseudotyped lentivirus, a codon-optimized cytomegalovirus/R-SARS-CoV-2 S (Wuhan-1, GenBank, MN908947.3) plasmid was constructed and subsequently modified via site-directed mutagenesis to contain the D614G mutation. Further mutations were integrated into the D614G background to recapitulate the spike mutations of both the B.1.1.7 α and B.1.351 β variants. GenBank ID QHD43416.1 was used as a reference sequence with the changes below:

B.1.1.7: H69del-V70del-Y144del-N501Y-A570D-D614G-P681H-T716I-S982A-D1118H and B.1.351: L18F-D80A-D215G-(L242-244)del-R246I-K417N-E484K-N501Y-D614G-A701V.

Pseudoviruses were produced by cotransfecting human embryonic kidney (HEK) 293T/17 cells [American Type Culture Collection (ATCC), CRL-11268] with plasmids encoding a luciferase reporter, a lentivirus backbone, and the SARS-CoV-2 S genes into HEK293T/17 cells as previously described (*82*). A human TM protease serine 2 (TMPRSS2) plasmid was cotransfected to produce pseudovirus (*83*). Neutralizing antibody responses in sera were assessed by pseudovirus neutralization assay as previously described (*3*, *22*). Briefly, heat-inactivated sera were serially diluted in duplicate, mixed with pseudovirus previously titrated to yield 10^4^ relative light units (RLU), and incubated at 37°C and 5% CO_2_ for about 45 min. 293T-hACE2.mF cells (courtesy of H. Mu and M. Farzan, The Scripps Research Institute) were diluted to a concentration of 7.5 × 10^4^ cells/ml in Dulbecco’s modified Eagle’s medium (DMEM) (Gibco) supplemented with 10% fetal bovine serum (FBS) and 1% penicillin/streptomycin and added to the sera-pseudovirus mixture. Seventy-two hours later, cells were lysed, and luciferase activity (in RLU) was measured using a SpectraMax (Molecular Devices) luminometer. Percent neutralization was normalized, considering uninfected cells as 100% neutralization and cells infected with pseudovirus alone as 0% neutralization. Fifty percent inhibitory dose (ID_50_) titers were determined using a log(agonist) versus normalized-response (variable slope) nonlinear regression model in GraphPad Prism.

### Authentic virus neutralization

For neutralization of authentic SARS-CoV-2 virus, an FRNT assay was performed as previously described (*84*). Plasma were serially diluted (threefold) in serum-free DMEM in duplicate wells and incubated with 100 to 200 focus-forming units of infectious clone-derived SARS-CoV-2-mNG virus (*85*) at 37°C for 1 hour. The antibody-virus mixture was added to VeroE6 cell (C1008, ATCC, #CRL-1586) monolayers seeded in 96-well blackout plates and incubated at 37°C for 1 hour. After incubation, the inoculum was removed and replaced with prewarmed complete DMEM containing 0.85% methylcellulose. Plates were incubated at 37°C for 24 hours. After 24 hours, the methylcellulose overlay was removed; cells were washed twice with PBS and fixed with 2% paraformaldehyde in PBS for 30 min at room temperature. After fixation, plates were washed twice with PBS, and foci were visualized on a fluorescence enzyme-linked immune absorbent spot reader (CTL ImmunoSpot S6 Universal Analyzer) and enumerated using Viridot (*86*). The neutralization titers were calculated as follows: 1 − (ratio of the mean number of foci in the presence of sera and foci at the highest dilution of respective sera sample). Each specimen was tested in two independent assays performed at different times. The FRNT-50% mNeonGreen (mNG_50_) titers were interpolated using a four-parameter nonlinear regression in GraphPad Prism. Samples with an FRNT-mNG_50_ value that was below the limit of detection were plotted at 10. For these samples, this value was used in fold reduction calculations.

### Quantification of antigen-specific B cells, intracellular cytokine staining, and flow cytometry

Cryopreserved PBMC were stained for antigen-specific B cells and subsets (fig. S6) using the following panels: IgM brilliant ultraviolet (BUV) 395 (1:50; clone G20-127, BD Biosciences), CD8 BUV665 (1:100; clone RPAT8, BD Biosciences), CD56 BUV737 (1:100; clone NCAM16, BD Biosciences), IgD fluorescein isothiocyanate (FITC) (1:33; SouthernBiotech), IgA Dy405 (1:167; polyclonal, Jackson ImmunoResearch), aqua LIVE/DEAD (1:400; Invitrogen), CD14 BV785 (1:200; clone M5E2, BioLegend), CD20 phycoerythrin (PE)–Alexa Fluor 700 (1:80; clone 2H7, Vaccine Research Center), IgG Alexa Fluor 700 (1:20; clone G18-145, BD Biosciences), and CD3 allophycocyanin (APC)–Cy7 (1:100; clone SP34-2, BD Pharmingen). Biotinylated prefusion-stabilized spike (S-2P) and spike subdomain (NTD and RBD) probes were produced as previously described (*81*) and conjugated to streptavidin-labeled dyes (BD Biosciences) to yield the following and streptavidin-conjugated B cell probes, NTD SA-BB700, RBD SA-BV650, and S-2P SA-APC. PBMCs were thawed into complete RPMI-1640 + 10% FBS (*87*), washed with PBS, and stained with an aqua LIVE/DEAD kit in PBS for 20 min at 4°C. Staining was then completed with the remainder of the antibody and probe cocktail described above in PBS for 45 min at 4°C. Samples were washed twice with PBS before flow cytometry.

To measure vaccine-specific T cell responses, cryopreserved PBMCs were thawed and rested overnight in a 37°C/5% CO_2_ incubator. The next morning, cells were stimulated with SARS-CoV-2 spike protein (S1 peptide pools, JPT Peptide Technologies Inc.) at a final concentration of 2 μg/ml in the presence of monensin and costimulatory antibodies anti-CD28 and anti-CD49d (clones CD28.2 and 9F10, BD Biosciences) for 6 hours. Negative controls received an equal concentration of dimethylsulfoxide (instead of peptides) and costimulatory antibodies. Intracellular cytokine staining and gating for CD4 and CD8 were performed as previously described (*88*) except the following monoclonal antibodies were added: PD-1 BUV737 (clone EH12.1, BD Biosciences) in place of PD-1 BV785, TNF-FITC (clone Mab11, BD Biosciences) in place of IL-5 BB515, and CD154 (CD40L) BV785 (clone 24-31, BioLegend). T_FH_ subsets were gated as CXCR5^+^, PD-1^+^, and ICOS^+^. An aqua LIVE/DEAD kit (Invitrogen) was used to exclude dead cells. All antibodies were previously titrated to determine the optimal concentration. All phenotyping and intracellular cytokine staining data were acquired on an BD FACSymphony flow cytometer and analyzed using FlowJo version 9.9.6 (Treestar Inc.).

### Luminex isotype and Fc receptor binding assay

To determine relative concentrations of antigen-specific antibody isotypes and Fc receptor binding activity in the rhesus samples, a customized Luminex isotype assay was performed as previously described (*89*). Antigens including SARS-CoV-2 full-length spike [provided by E. Fischer, Dana-Farber Cancer Institute (DFCI)] and RBD (provided by A. Schmidt, Ragon Institute) were covalently coupled to Luminex MicroPlex carboxylated bead regions (Luminex Corporation) using *N*-hydroxysuccinimide (NHS) ester linkages with Sulfo-NHS and EDC (Thermo Fisher Scientific) according to the manufacturer’s recommendations. Immune complexes were formed by incubating antigen-coupled beads with diluted samples while rotating overnight. Mouse anti-rhesus antibody detectors were then added for each antibody isotype (IgG1, IgG2, IgG3, IgG4, and IgA; NIH Nonhuman Primate Reagent Resource supported by AI126683 and OD010976). Then, tertiary anti-mouse IgG detector antibodies conjugated to PE were added. Flow cytometry was performed using an iQue Plus Screener (Intellicyt) with a robot arm (PAA Automation). Analysis of flow cytometry data was performed using iQue Intellicyt software.

### Systems serology

To quantify antibody functionality of plasma samples, bead-based assays were used to measure antibody-dependent cellular phagocytosis (ADCP), antibody-dependent neutrophil phagocytosis (ADNP), and antibody-dependent complement deposition (ADCD), as previously described (*90*–*92*). SARS-CoV-2 spike protein (provided by E. Fischer, DFCI) was coupled to fluorescent streptavidin beads (Thermo Fisher Scientific) and incubated with diluted plasma samples to allow antibody binding to occur. For ADCP, cultured human monocytes (THP-1 cell line, ATCC) were incubated with immune complexes to induce phagocytosis. For ADNP, primary PBMCs were isolated from whole blood from blood bank–sourced healthy donors using an ammonium-chloride-potassium lysis buffer. After phagocytosis of immune complexes, neutrophils were stained with an anti-CD66b Pacific Blue detection antibody (BioLegend) as previously described (*90*–*92*). For detection of complement deposition, lyophilized guinea pig complement (Cedarlane) was reconstituted according to the manufacturer’s instructions and diluted in a gelatin veronal buffer with calcium and magnesium (Boston BioProducts). After ADCD occurred, C3 bound to immune complexes was detected with the FITC-Conjugated Goat IgG Fraction to Guinea Pig Complement C3 (MP Biomedicals) as previously described (*90*–*92*).

For quantification of antibody-dependent natural killer (NK) cell activation (*93*), diluted plasma samples were incubated in Nunc MaxiSorp plates (Thermo Fisher Scientific) coated with antigen. Human NK cells were isolated the evening before using the RosetteSep Human NK Cell Enrichment Cocktail (STEMCELL Technologies) from healthy buffy coat donors and incubated overnight with human recombinant IL-15 (STEMCELL Technologies). As previously described (*93*), NK cells were incubated with immune complexes, CD107a PE-Cy5 (BD Biosciences), Golgi stop (BD Biosciences), and brefeldin A (Sigma-Aldrich). After incubation, cells were stained using anti-CD16 APC-Cy7 (BD Biosciences), anti-CD56 PE-Cy7 (BD Biosciences), and anti-CD3 Pacific Blue (BD Biosciences) and then fixed (Perm A, Life Technologies). Intracellular staining was done with anti–IFN-γ FITC (BD Biosciences) and anti–macrophage inflammatory protein 1β (MIP-1β) PE (BD Biosciences) after permeabilizing the NK cells with Perm B (Thermo Fisher Scientific). Flow cytometry acquisition of all assays was performed using the iQue Screener Plus (Intellicyt) and an S-LAB robot (PAA). For ADCP, phagocytosis events were gated on bead-positive cells. For ADNP, neutrophils were identified by gating on CD66b^+^ cells, and phagocytosis was identified by gating on bead-positive cells. A phagocytosis score for ADCP and ADNP was calculated as (percentage of bead-positive cells) × [median fluorescence intensity (MFI) of bead-positive cells] divided by 10,000. ADCD quantification was reported as MFI of FITC–anti-C3. For antibody-dependent NK activation, NK cells were identified by gating on CD3^−^, CD16^+^, and CD56^+^ cells. Data were reported as the percentage of cells positive for CD107a, IFN-γ, and MIP-1β.

### Quantification of sgRNA after challenge

Nasal swabs and BAL fluid were collected 2, 4, and 7 days after challenge. At the time of collection, nasal swabs were frozen in 1 ml of PBS containing 1 μl of SUPERase-In RNase Inhibitor (Invitrogen) and frozen at −80°C until extraction. Nasal specimens were thawed at 55°C, and the swab was removed. The remaining PBS was mixed with 2 ml of RNAzol BD (Molecular Research Center) and 20 μl of acetic acid. At the time of collection, 1 ml of BAL fluid was mixed with 1 ml of RNAzol BD containing 10 μl of acetic acid and frozen at −80°C until extraction. BAL specimens were thawed at room temperature and mixed with an additional 1 ml of RNAzol BD containing 10 μl of acetic acid.

Total RNA was extracted from nasal specimens and BAL fluid using RNAzol BD Column Kits (Molecular Research Center) and eluted in 65 μl of H_2_O. Subgenomic SARS-CoV-2 E (envelope) mRNA was quantified via PCR using a technique similar to that described previously (*1*). Reactions were conducted with 5 μl of RNA and TaqMan Fast Virus 1-Step Master Mix (Applied Biosystems) with 500 nM primers and 200 nM probes. Primers and probes were as follows: sgLeadSARSCoV2_F (5′-CGATCTCTTGTAGATCTGTTCTC-3′), E_Sarbeco_P (5′-FAM-ACACTAGCCATCCTTACTGCGCTTCG-BHQ1-3′), and E_Sarbeco_R (5′-ATATTGCAGCAGTACGCACACA-3′).

Reactions were run on the QuantStudio 6 Pro Real-Time PCR System (Applied Biosystems) at the following conditions: 50°C for 5 min, 95°C for 20 s, and 40 cycles of 95°C for 15 s and 60°C for 1 min. Absolute quantification was performed in comparison to a standard curve. For the standard curve, the E subgenomic mRNA sequence was inserted into a pcDNA3.1 vector (GenScript) and transcribed using the MEGAscript T7 Transcription Kit (Invitrogen) followed by the MEGAclear Transcription Clean-Up Kit (Invitrogen). The lower limit of quantification was 50 copies.

### Histopathology

Histopathological analyses were performed as described previously (*27*). Immunohistochemistry (IHC) was used to visualize SARS-CoV-2 nucleocapsid antigen (rabbit polyclonal, GeneTex) and eosinophils by staining for eosinophil peroxidase (rabbit polyclonal, Atlas Antibodies). Inflammation was scored on the following scale based on the percentage of tissue affected: 0, 0%; 1, <10%; 2, 10 to 25%; 3, 26 to 50%; and 4, >50%. Viral antigen as observed by IHC was scored on the following scale: 0, minimal to absent; 1, minimally abundant but clearly present; 2, mildly abundant; 3, moderately abundant; and 4, abundant. Eosinophils as observed by IHC were scored on the following scale: 0, within normal limits; 1, minimal increase; 2, mild increase; 3, moderate increase; and 4, abundant.

### Statistics

All statistical analyses were performed in GraphPad Prism version 8.4. For comparisons between NHP vaccine groups at a single time point or dilution, a Kruskal-Wallis test with Dunn’s multiple comparisons test was performed. For comparisons over a time course, a two-way analysis of variance (ANOVA) was used with the Geisser-Greenhouse correction and the Tukey test to correct for multiple comparisons (Sidak’s test was used for comparing only two groups over a dilution series). For T cell assays, a mixed-effect model with the Geisser-Greenhouse correction and a Tukey test was used, because not all samples were available at each time point. For comparisons of viral load in NHP and viral load and weight loss in hamsters over time, a two-way ANOVA was used with the Geisser-Greenhouse correction and a Dunnett test to correct for multiple comparisons, with comparisons to the PBS control group only. For comparisons of NHP titers postchallenge over time (not all samples were available at the last time point), a mixed-effect model was used with the Geisser-Greenhouse correction and a Dunnett test, with comparisons controlled to the week 5 time point. For the weight loss versus binding titer correlation, a Spearman correlation test was performed. All binding titer and viral load data were log-transformed before performing statistical tests.

## Notes

View/request a protocol for this paper from *Bio-protocol*.
