# Supplementary material for: Protective antibodies elicited by SARS-CoV-2 spike protein vaccination are boosted in the lung after challenge in nonhuman primates
Source: Sci Transl Med. 2021 Jul 27;13(607):eabi4547. doi: 10.1126/scitranslmed.abi4547 (PMC9266840; doi:10.1126/scitranslmed.abi4547)
Supplement: Supplementary file 1 — Figs. S1 to S6 [file scitranslmed.abi4547_sm.pdf]

Science Translational Medicine
AAAS

Supplementary Materials for

Protective antibodies elicited by SARS-CoV-2 spike protein vaccination are boosted in the lung after challenge in nonhuman primates

Joseph R. Francica *et al.*

Corresponding author: Robert A. Seder, rseder@mail.nih.gov

Sci. Transl. Med. **13**, eabi4547 (2021)
DOI: 10.1126/scitranslmed.abi4547

The PDF file includes:

Figs. S1 to S6

Other Supplementary Material for this manuscript includes the following:

Data files S1 to S4

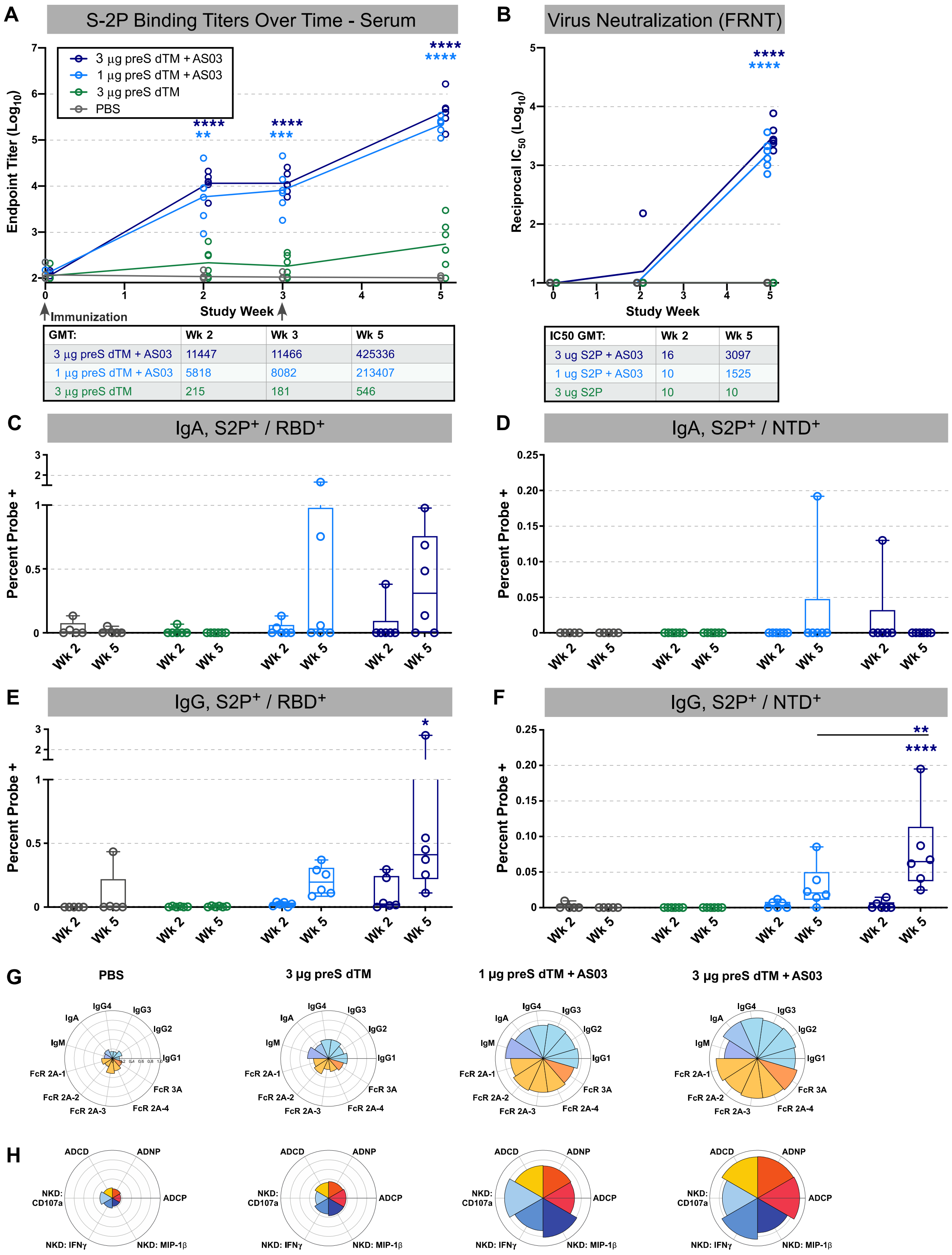

GMT:	Wk 2	Wk 3	Wk 5
3 µg preS dTM + AS03	11447	11466	425336
1 µg preS dTM + AS03	5818	8082	213407
3 µg preS dTM	215	181	546

IC50 GMT:	Wk 2	Wk 5
3 ug S2P + AS03	16	3097
1 ug S2P + AS03	10	1525
3 ug S2P	10	10

Fig. S1. AS03 adjuvant is required for functional antibody responses.
Rhesus macaques (n=6 per group) were immunized at weeks (Wk) 0 and 3 with 1 or 3 μg of transmembrane-deleted prefusion spike (preS dTM) protein adjuvanted with AS03 adjuvant or 3 μg of preS dTM formulated with PBS only. (**A**) Endpoint binding titers over time following vaccination. (**B**) Live virus neutralization over time; 50% inhibitory concentration (IC_{50}) values are plotted. Symbols represent individual animals; bars and lines indicate group geometric means, and geometric mean titers (GMT) are indicated in table below. FRNT, focus reduction neutralization test. (**C to F**) Antigen-specific B cells were enumerated in the peripheral blood mononuclear cell (PBMC) compartment using S-2P, receptor binding domain (RBD) and N-terminal domain (NTD) B cell probes after prime (week 2) and boost (week 5). IgA vaccine-specific cells were found to bind both the S-2P and RBD probes (**C**); and S-2P and NTD probes, (**D**). IgG vaccine-specific cells were found to bind both the S-2P and RBD probes (**E**); and S-2P and NTD probes, (**F**). Symbols represent individual animals; box plots indicate the median and interquartile range; whiskers indicate minimum and maximum data points. (**G and H**) A systems serology approach was used to measure antibody subtypes, isotypes and F_C receptor (F_CR) binding (**G**), as well as F_C-mediated effector functions (**H**) using spike protein. Flower plots indicate a normalized Z-score for each parameter. ADCD, antibody dependent complement deposition; ADNP, antibody dependent neutrophil phagocytosis; ADCP, antibody dependent cellular phagocytosis; NKD, natural killer cell degranulation; IFN-γ, interferon gamma; macrophage inflammatory protein, MIP-1β. Asterisks indicate significance compared to the PBS control group at each time point (unless otherwise indicated): *, p<0.05; **, p<0.01; ***, p<0.001 ****, p<0.0001.

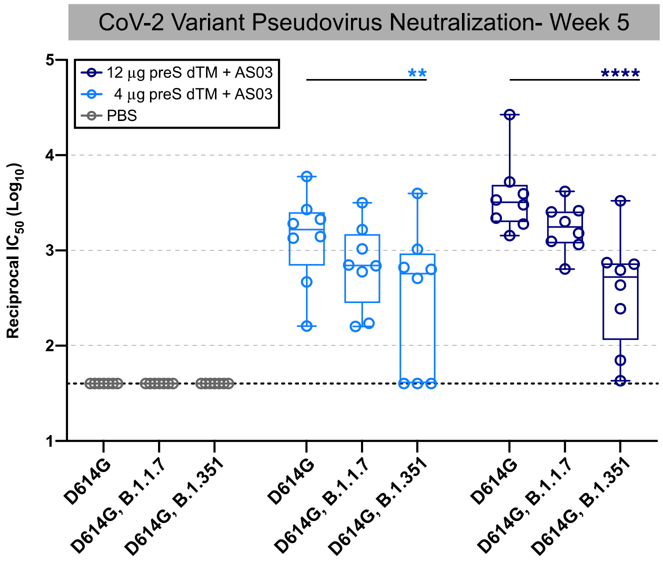

Endpoint GMT:	D614G	D614G, B.1.1.7	D614G, B.1.351
12 µg preSΔTM + AS03	3671	1719	370
4 µg preSΔTM + AS03	1319	674	294

Fig. S2. Pseudoviral neutralization of SARS-CoV-2 variants.
Pseudoviral neutralization responses in rhesus macaques immunized with 4 or 12 ug of preS dTM adjuvanted with AS03 adjuvant at weeks 0 and 3. Plasma from week 5 were tested against the parental D614G virus, or against B.1.1.7 (α variant) or B.1.351 (β variant) also containing the D614G mutation. Geometric mean titers (GMTs) for neutralization are indicated in the table below the graph. Symbols represent individual animals; box plots indicate the median and interquartile range; whiskers indicate minimum and maximum data points. Asterisks indicate significance compared to the D614G virus for each vaccine group. **, $p<0.01$; ****, $p<0.0001$.

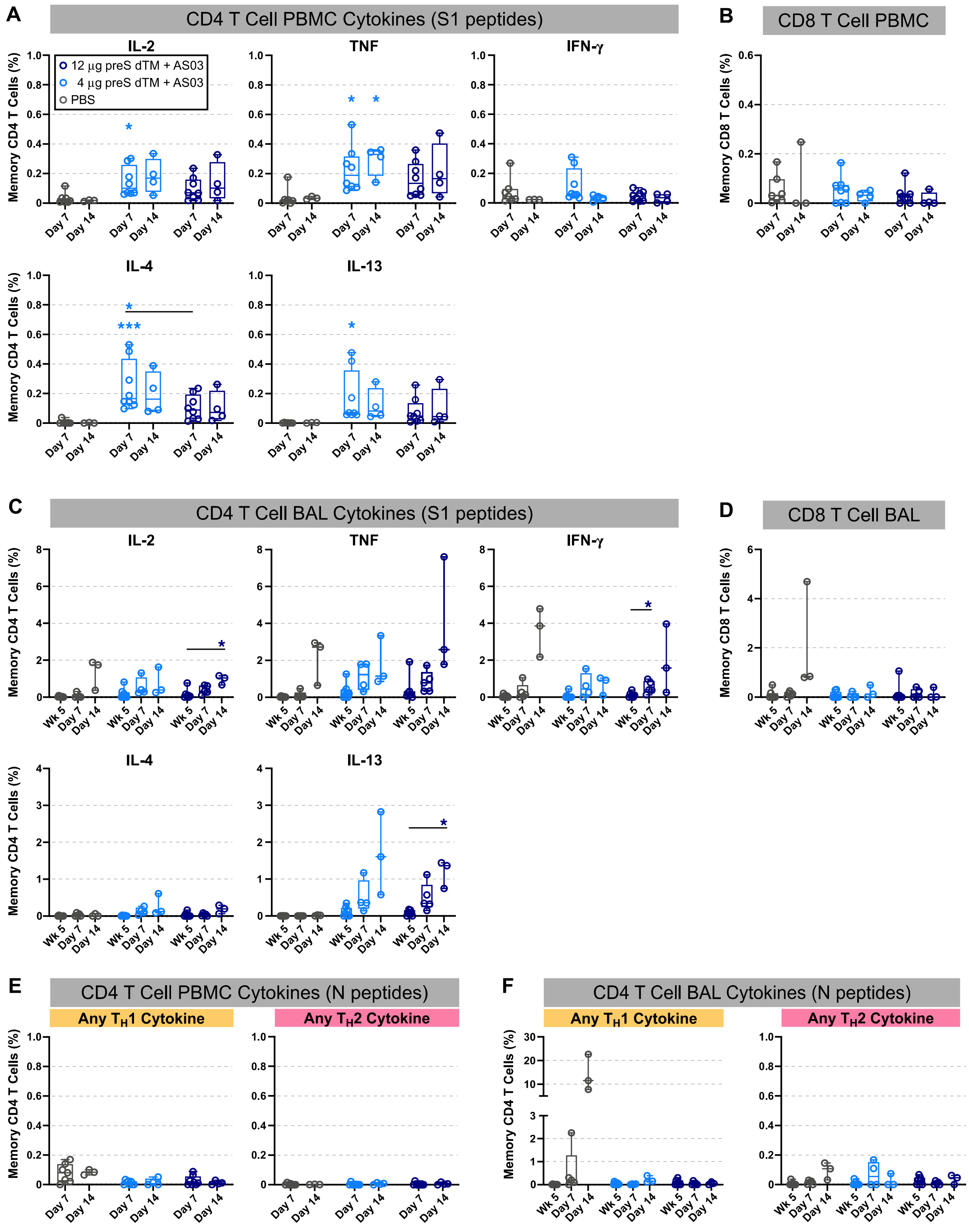

A
CD4 T Cell PBMC Cytokines (S1 peptides)
IL-2
TNF
IFN-γ
12 µg preS dTM + AS03
4 µg preS dTM + AS03
PBS
Memory CD4 T Cells (%)
IL-4
IL-13
B
CD8 T Cell PBMC
Memory CD8 T Cells (%)
C
CD4 T Cell BAL Cytokines (S1 peptides)
D
CD8 T Cell BAL
E
CD4 T Cell PBMC Cytokines (N peptides)
Any TH1 Cytokine
Any TH2 Cytokine
F
CD4 T Cell BAL Cytokines (N peptides)
Wk 5
Day 7
Day 14

Fig. S3. T cell responses following SARS-CoV-2 challenge.

PBMC and bronchoalveolar lavage (BAL) cells were collected prior to challenge (week 5) and on days 7 and 14 following SARS-CoV-2 challenge. Cells were stimulated with pools of peptides covering the spike (S1) or nucleocapsid (N) proteins. (**A**) Percent of PBMC memory CD4 T cells making the indicated cytokines following stimulation with S1 peptides. (**B**) Percent of memory PBMC CD8 T cells making any T_H1 cytokine following stimulation with S1 peptides. (**C**) Percent of memory BAL CD4 T cells making the indicated cytokines following stimulation with S1 peptides. (**D**) Percent of BAL memory CD8 T cells making any T_H1 cytokine following stimulation with S1 peptides. (**E**) Percent of PBMC memory CD4 T cells making any T_H1 (left graph) or any T_H2 cytokine (right graph) following stimulation with N peptides. (**F**) Percent of BAL memory CD4 T cells making any T_H1 (left graph) or any T_H2 cytokine (right graph) following stimulation with N peptides. Symbols represent individual animals; box plots indicate the median and interquartile range; whiskers indicate minimum and maximum data points. Asterisks indicate significance compared to the PBS control group for each time point (unless otherwise indicated) as follows: *, $p<0.05$; ***, $p<0.001$.

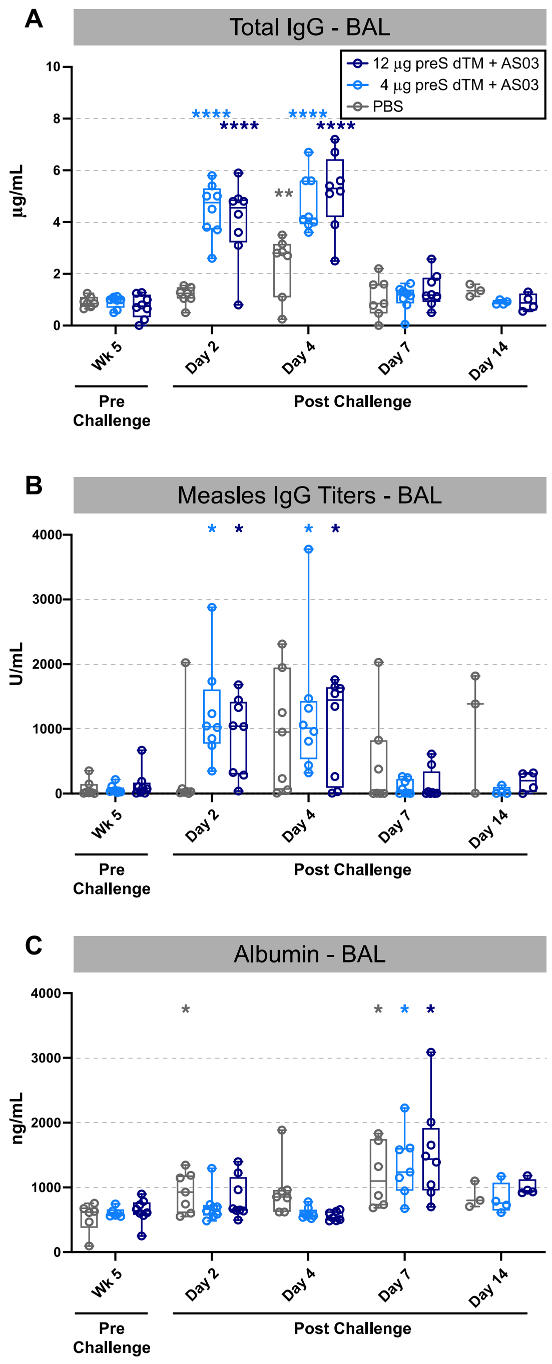

Fig. S4. Nonspecific antibody responses were observed in the lung following SARS-CoV-2 challenge.

Bronchoalveolar lavage (BAL) supernatant was collected prior to challenge (week 5) and on days 2, 4, 7 and 14 following SARS-CoV-2 challenge. (**A**) Total IgG concentration titers in BAL were measured. (**B**) Measles-specific IgG responses in BAL are shown. (**C**) Albumin concentrations in BAL were measured. Symbols represent individual animals; box plots indicate the median and interquartile range; whiskers indicate minimum and maximum data points. Asterisks indicate significance compared to the week 5 time point as follows: *, $p<0.05$; **, $p<0.01$; ****, $p<0.0001$.

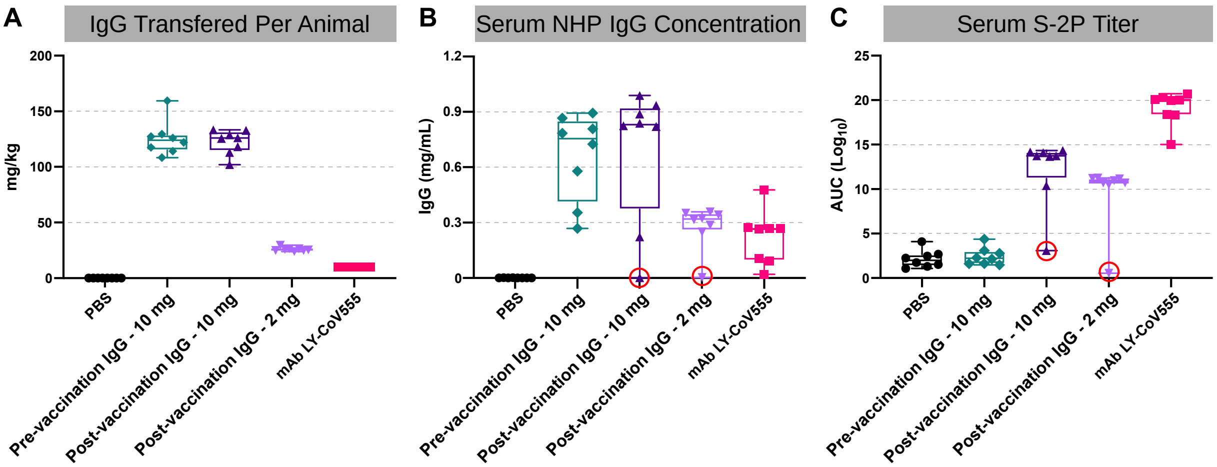

Fig. S5. Quantification of IgG following passive transfer from vaccinated NHP to hamsters. Total IgG was isolated from rhesus macaques immunized with 3 µg of AS03-adjuvanted preS dTM. Ten or 2 mg total IgG was transferred to hamsters, or PBS was given as a negative control; serum was collected one day later, on the day of challenge. (**A**) The amount of IgG transferred based on the weight of each animal is shown; the mAb Ly-CoV555 was delivered at 10 mg/kg. (**B**) An NHP total IgG ELISA was used to measure the concentration of NHP IgG in hamster serum by extrapolating from an NHP IgG standard curve. (**C**) S-2P ELISA reactivity of hamster serum is shown as area under the curve (AUC) values. Red circles indicate 2 animals that were improperly infused and were thus excluded from the weight loss analysis in Figure 6B. Symbols represent individual animals; box plots indicate the median and interquartile range; whiskers indicate minimum and maximum data points.

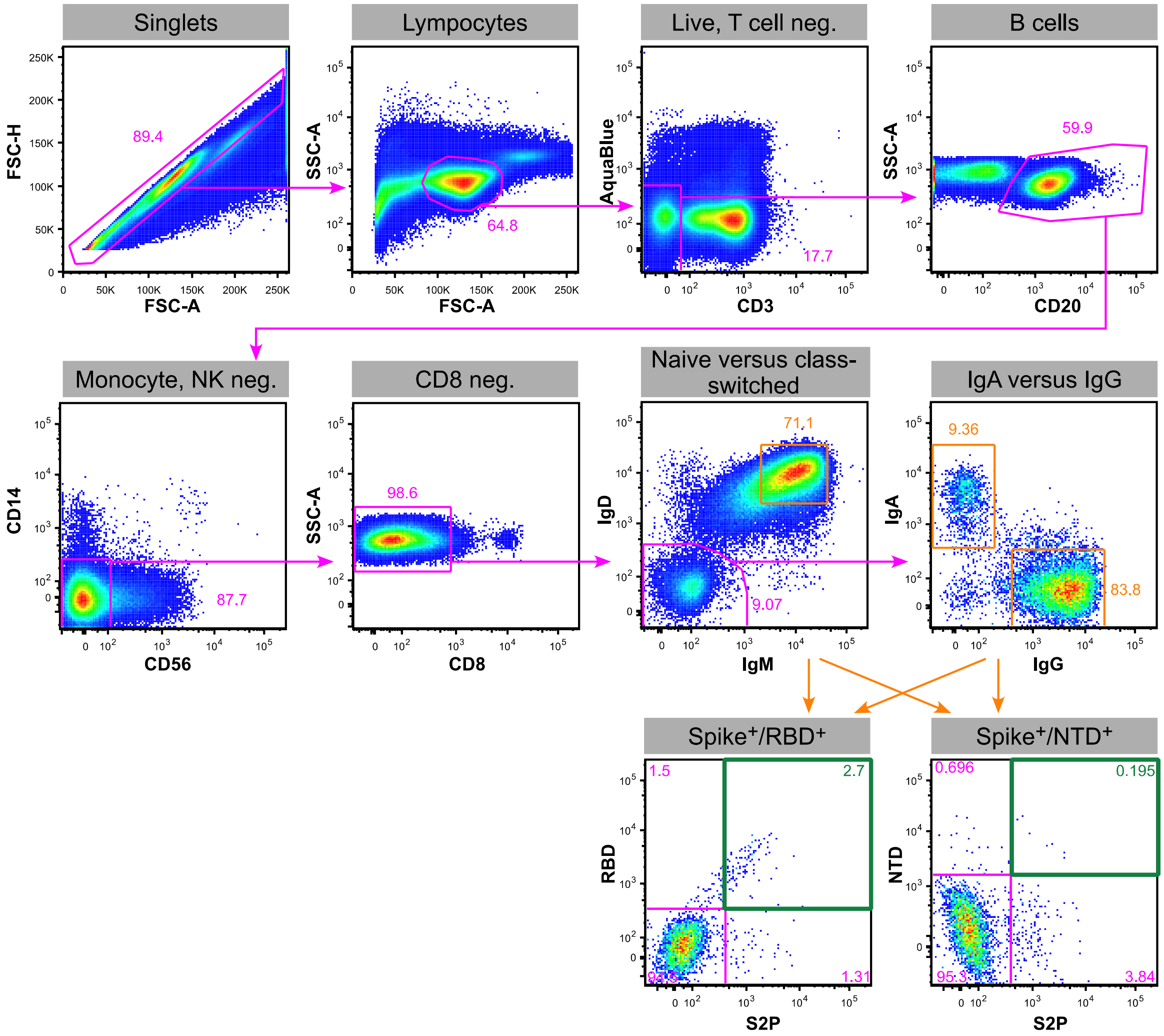

Fig. S6. Gating tree for isotyping antigen-specific B cells.
Vaccine-specific B cells were identified from PBMC by first gating on singlets based on forward scatter height (FSC-H) and area (FSC-A), followed by gating for lymphocytes based on FSC-A and side scatter area (SSC-A). B cells were then negatively gated for CD3 and viability dye, positively gated on CD20, then negatively gated on CD56 to exclude natural killer (NK) cells, CD14 to exclude monocytes and CD8. Class-switched (memory) B cells were gated as IgD and IgM negative; double-positive cells in this plot were gated as naïve B cells. Memory B cells were then further separated by IgA and IgG markers, followed by gating for SARS-CoV-2 spike antigen-specific cells by gating for cells that were positive for both S-2P and RBD, or S-2P and NTD probes (green boxes).
